# Association between nocturnal ozone enhancement and daily cardiovascular mortality: A multi-city study in China

**DOI:** 10.1016/j.eehl.2025.100211

**Published:** 2025-12-24

**Authors:** Zhihan Jian, Peng Yin, Renjie Chen, Lijun Wang, Yixiang Zhu, Xia Meng, Haidong Kan, Yue Niu, Maigeng Zhou

**Affiliations:** a School of Public Health, Key Lab of Public Health Safety of the Ministry of Education, NHC Key Lab of Health Technology Assessment, Fudan University, Shanghai 200032, China; b National Center for Chronic and Noncommunicable Disease Control and Prevention, Chinese Center for Disease Control and Prevention, Beijing 102206, China

**Keywords:** Nocturnal ozone enhancement, Mortality, Cardiovascular disease, Time series

## Abstract

The health effects of ambient ozone (O_3_) pollution have been well documented, but most studies have focused on daytime exposure, with limited research on nighttime O_3_. Nocturnal O_3_ enhancement (NOE) refers to unexpected increases in nighttime O_3_ concentrations over a few hours, yet the health effects of this phenomenon remain unknown. This study evaluated the short-term association between NOE days and cardiovascular mortality by conducting a multi-city time-series analysis in China (2013–2015). Nine definitions were adopted to identify site-level NOE events, considering both average O_3_ levels and peak increments at night. A city-level NOE day was defined as a day when over 60% of the monitoring stations within a city recorded at least one site-level NOE event. City-level associations were analyzed using over-dispersed generalized additive models, and national estimates were pooled using meta-analysis. We also conducted stratified analyses by age, sex, season, and region. We found significant associations between NOE days and increased mortality due to total cardiovascular disease (CVD) and coronary heart disease (CHD). Under the strictest NOE definition, mortality risk increased by 1.7% (95% CI: 0.8%, 2.5%) for total CVD and 2.1% (95% CI: 0.9%, 3.3%) for CHD on the day after NOE. Relatively higher risk estimates were found in individuals aged 5–64 years, females, the warm season, and the southern region. No stable associations were found with stroke mortality. These findings offer new insights into the health impacts of NOE and underscore the need for time-phased actions to control both daytime and nighttime O_3_ air pollution.

## Highlights

• Nocturnal ozone enhancement was associated with elevated cardiovascular mortality.
• The effects of nocturnal ozone enhancement were independent from daytime ozone exposure.
• The associations were stronger in individuals aged 5–64 years, females, the warm season, and the southern region.

## Graphical abstract

**Figure:**
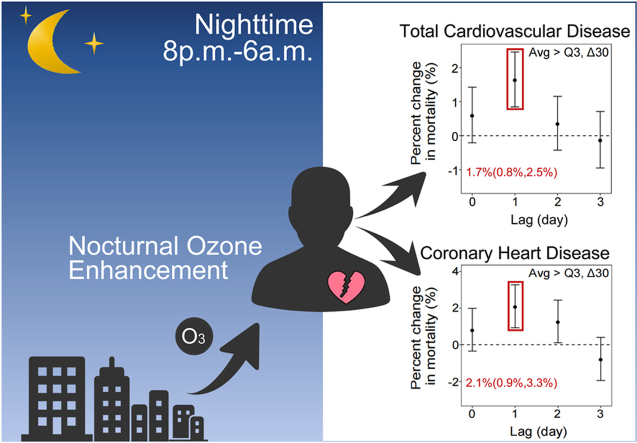

## 1 Introduction

Tropospheric ozone (O_3_) is primarily formed through photochemical reactions between nitrogen oxides (NO_x_) and volatile organic compounds (VOCs) under ultraviolet radiation [1]. Therefore, unlike other air pollutants, e.g., fine particulate matter (PM_2.5_), the concentration of O_3_ exhibits a distinct diurnal pattern, being low in the early morning, peaking in the late afternoon, and then gradually declining overnight. However, recent studies have documented nocturnal O_3_ enhancement (NOE) events, i.e., unexpected increases in O_3_ levels at night, in several regions [[2], [3], [4]]. These events may be caused by vertical mixing in the boundary layer, unstable nocturnal atmospheric conditions, or specific meteorological conditions such as strong vertical wind shear and low-level jets [5,6]. Recent research has shown that NOE events occurred nearly 1.5 times more frequently in China than in Europe and the United States [4]. Given that NOE events may prolong O_3_ exposure duration and increase total O_3_ exposure levels, further investigation into their impacts is needed.

Short-term O_3_ exposure has been linked to increased cardiovascular disease (CVD) mortality and morbidity in numerous epidemiological studies [1,7,8]. These studies typically used metrics such as daily 24-h average concentrations, maximum daily 1-h average (MDA1) concentrations, and maximum daily 8-h average (MDA8) concentrations to assess short-term O_3_ exposure [9,10]. For example, our previous study reported that each 10 μg/m^3^ increment in MDA8 O_3_ concentrations was associated with a 0.27% rise in total CVD mortality [8]. Overall, existing epidemiological studies have focused predominantly on daytime O_3_ pollution, while the adverse health effects of nighttime O_3_ pollution have been understudied, largely due to the prevailing assumption that O_3_ concentrations drop to extremely low levels overnight. However, recent observations of frequent NOE events have challenged this notion. Research on the health effects of NOE events is urgently needed to inform time-phased O_3_ control strategies in the future. Notably, researchers have observed stronger associations of MDA1 and MDA8 O_3_ concentrations with health outcomes compared to daily 24-h average O_3_ concentrations [9,10]. Controlled human exposure studies also indicated that, at equivalent cumulative exposure levels, peak O_3_ exposure may lead to greater adverse effects than exposure to a constant concentration [11,12]. This implies that peak O_3_ levels over several hours may better capture health risks than averages. Moreover, there is biological plausibility for the impact of nighttime O_3_ exposure on cardiovascular health. Multiple cardiovascular physiological processes exhibit circadian rhythms, with parasympathetic activity predominating at night to facilitate cardiac recovery [[13], [14], [15]]. Previous studies have shown that even low levels of O_3_ exposure may activate the sympathetic nervous system [16,17], potentially disrupting cardiac recovery at night and affecting cardiovascular function. Therefore, we hypothesized that, similar to daytime O_3_ peaks, NOE events may also have potential effects on cardiovascular health.

To test this hypothesis, we investigated the associations between NOE and cardiovascular mortality using an established multi-city time-series dataset in China. Additionally, we assessed whether these associations were modified by age, sex, season, and region.

## 2 Material and methods

### 2.1 Study design and death data collection

Daily mortality data were obtained from the Disease Surveillance Points system administered by the Chinese Center for Disease Control and Prevention. The system was subject to strict multi-level quality control to ensure the completeness of death records, as detailed in previous publications [18]. Causes of death were coded according to the International Classification of Diseases, 10th revision (ICD-10). In this study, we extracted data on deaths from total CVD (ICD-10 code: I00-I99), coronary heart disease (CHD, ICD-10 code: I20-I25), and stroke (ICD-10 code: I60-I69), with no missing data.

Hourly PM_2.5_ and O_3_ concentration data were obtained from China’s National Urban Air Quality Real-time Publishing Platform (https://air.cnemc.cn:18007/). Using these measurements, we calculated daily 24-h average PM_2.5_ concentrations, 24-h average O_3_ concentrations, MDA8 O_3_ concentrations, and nighttime average O_3_ concentrations only when at least 75% of the hourly measurements were available for a given period. Consistent with previous studies conducted in China, MDA8 O_3_ was defined as the average concentration between 10:00 a.m. and 6:00 p.m., as O_3_ typically reaches its peak during this period [8,19,20]. Nighttime average O_3_ concentrations were calculated based on measurements between 8:00 p.m. and 6:00 a.m. The percentages of missing data for 24-h average PM_2.5_, 24-h average O_3,_ MDA8 O_3_, and nighttime average O_3_ were 8.56%, 10.17%, 6.95%, and 11.95%, respectively. Additionally, daily mean temperature and relative humidity data were acquired from the China Meteorological Data Sharing Service System (http://data.cma.cn/).

### 2.2 Definition of nocturnal ozone enhancement

Due to the lack of a standardized definition for NOE events, we adopted multiple criteria in line with previous studies [6,21,22]. Given that the health effects of O_3_ depend on both average and peak concentrations, we defined NOE events by considering the nighttime average O_3_ concentrations (8:00 p.m. to 6:00 a.m.) and the difference between any two consecutive hourly O_3_ concentrations during this period. For the former criterion, we established three thresholds where the nighttime average O_3_ concentration for a given day exceeded the monthly 25th, 50th, and 75th percentiles, abbreviated as “Avg > Q1”, “Avg > Q2”, and “Avg > Q3”, respectively. For the latter criterion, the difference between consecutive hourly O_3_ concentrations exceeding 10 μg/m^3^ (abbreviated as “Δ10”) was used to define NOE events, as in previous studies [6,21]. We also used increment thresholds of 20 μg/m^3^ and 30 μg/m^3^ (abbreviated as “Δ20” and “Δ30”) to capture larger peaks in NOE events. By combining these two criteria, we consequently generated nine NOE definitions (Table 1), with the definition of “Avg > Q3, Δ30” being the strictest and the definition of “Avg > Q1, Δ10” being the most lenient.

**Table 1:** Summary descriptive statistics of definitions and numbers of days with nocturnal ozone enhancement at the city level in 272 Chinese cities from 2013 to 2015.

Definition	Description		Number of days with NOE per year							Total number of days with NOE
	Average (Avg)	Difference (Δ)	Mean	SD	Min	P_25_	Median	P_75_	Max	
Avg > Q1, Δ10	>quartile 1	>10 μg/m^3^	151	50	8	119	147	184	261	75,775
Avg > Q1, Δ20	>quartile 1	>20 μg/m^3^	80	52	1	39	72	110	258	39,818
Avg > Q1, Δ30	>quartile 1	>30 μg/m^3^	43	42	0	14	31	63	251	21,626
Avg > Q2, Δ10	>quartile 2	>10 μg/m^3^	109	32	4	88	108	131	176	54,631
Avg > Q2, Δ20	>quartile 2	>20 μg/m^3^	62	36	1	34	58	83	173	30,901
Avg > Q2, Δ30	>quartile 2	>30 μg/m^3^	35	31	0	12	27	53	169	17,577
Avg > Q3, Δ10	>quartile 3	>10 μg/m^3^	61	16	3	50	61	72	93	30,411
Avg > Q3, Δ20	>quartile 3	>20 μg/m^3^	37	19	1	23	36	49	89	18,571
Avg > Q3, Δ30	>quartile 3	>30 μg/m^3^	23	18	0	8	19	33	88	11,209

NOE, nocturnal ozone enhancement; SD, standard deviation; Min, minimum; P_25_, 25th percentile value; P_75_, 75th percentile value; Max, maximum.

After establishing nine NOE definitions, we first applied these definitions to define site-level NOE events at individual fixed-site monitoring stations, allowing for multiple NOE events to be recorded at a given station on the same night. Then, we classified a city-level NOE day as a day when more than 60% of its monitoring stations recorded at least one site-level NOE event on the same night [22]. By this approach, each day in each city was classified as either an NOE day or a non-NOE day.

To analyze the lagged associations between city-level NOE days and cardiovascular mortality, we defined several lag periods, including the day of death (lag 0 d), 1 day prior to death (lag 1 d), 2 days prior to death (lag 2 d), and 3 days prior to death (lag 3 d). For example, if a death occurred on June 2, 2015, the NOE day between 8:00 p.m. on June 1 and 6:00 a.m. on June 2 was defined as an NOE day at lag 0 d (Fig. S1).

### 2.3 Statistical analysis

This study employed a two-stage analytical approach to evaluate the associations between NOE days and cardiovascular mortality. In stage one, a generalized additive model with a quasi-Poisson family was applied to separately estimate the associations between NOE days and mortality due to total CVD, CHD, and stroke in each city. The dependent variable was the daily death counts, and the independent variable was a binary indicator for NOE days (1 for NOE days and 0 for non-NOE days). Differently defined NOE days at different lags were introduced into the model individually to avoid multicollinearity. The model also included: (1) a natural cubic spline of calendar day with 6 degrees of freedom (*df*) per year to account for seasonal and long-term trends; (2) a natural cubic spline of current-day relative humidity and temperature with 3 *df* to adjust for the nonlinear effects of meteorological conditions; (3) day of the week; and (4) MDA8 O_3_ concentrations to adjust for the impact of daytime O_3_ levels. The lag for MDA8 O_3_ was consistent with that for NOE days. In stage two, we conducted a meta-analysis to derive national-level associations. Potential heterogeneity between cities was assessed using *I*-square (*I*^2^) statistics from the Cochran’s Q test, with heterogeneity categorized as low (*I*^2^ < 25%), moderate (25% ≤ *I*^2^ ≤50 %), or high (*I*^2^ > 50%) [23]. To directly compare the effects of nighttime and daytime O_3_ exposure on total CVD mortality, we replaced the binary NOE indicator in the main analysis with nighttime O_3_ average concentrations and reanalyzed the data following the two-stage analytical approach.

We conducted several sensitivity analyses. First, we used different metrics to control for the effects of average or daytime O_3_ exposure by replacing single-day MDA8 O_3_ concentrations in the main analysis with either 4-day moving average MDA8 O_3_ concentrations (lag 0–3 d) or single-day 24-h averages. Second, we applied three alternative approaches to adjust for temperature: (1) changing the *df* for the natural spline from 3 to 6; (2) using a distributed lag nonlinear model (DLNM) to account for both nonlinear and lagged effects of temperature; and (3) incorporating a DLNM term of temperature and a binary variable for extreme heat simultaneously. In the DLNM, we applied a B-spline with 3 *df* for temperature and a natural cubic spline with two knots equally placed on the logarithmic values of the lag of 21 days. Consistent with previous studies [24,25], extreme heat was defined as days when the daily maximum temperature exceeded the 95th percentile of the city-specific temperature distribution. Third, we further adjusted for 4-day moving averages of PM_2.5_ concentrations in the main model and the model that adjusted temperature using DLNM. Fourth, we changed the *df* for the spline of calendar day from 6 to 5 or 7 per year. Finally, we restricted our analysis to 69 cities with 3 years of data.

In addition, we performed stratified analyses by age (5–64, 65–74, and ≥75 years), sex (male and female), region (north and south), and season (warm and cool) to characterize subgroup-specific associations. The southern and northern regions were divided based on the Qinling-Huaihe Line. The warm season was defined as May through October, and the cool season was defined as November through April. Due to limited daily CHD and stroke deaths, stratified analyses were confined to total CVD deaths. Between-group differences were evaluated using Cochran’s Q test.

All statistical analyses were conducted in R software (version 3.3.1), with the “mgcv” package for generalized additive models and the “mixmeta” package for meta-analysis and Cochran’s Q tests. *P*-values < 0.05 were considered statistically significant.

## 3 Results

### 3.1 Descriptive statistics of NOE days and cardiovascular death

Throughout the study period, a total of 1,715,918 people died from total CVD, including 642,476 deaths from CHD and 826,436 deaths from stroke (Table S1). The frequency of NOE days varied significantly by definition, season, and region. The strictest definition revealed 11,209 NOE days in 272 cities over the 3-year study period, while the most lenient definition identified 75,775 NOE days (Table 1). Seasonally, the total number of NOE days in the warm season (N = 5888) was 1.11 times that in the cool season (N = 5321) under the strictest definition (Table S2). Regionally, the total number of NOE days in the northern region (N = 7876) was 2.36 times that in the southern region (N = 3333) under the strictest definition (Table S3). Geographic variation was pronounced, with Kashi in Xinjiang Uygur autonomous region having the highest number of NOE days (88 days per year), whereas Yingtan in Jiangxi province, Shaoguan in Guangdong province, and Dali in Yunnan province had no NOE days throughout the year. In addition, throughout this period, the average 24-h PM_2.5_ concentration and MDA8 O_3_ concentration were 56 μg/m^3^ and 77 μg/m^3^, respectively (Table S1).

### 3.2 Associations between NOE and total CVD mortality

**Fig. 1:**
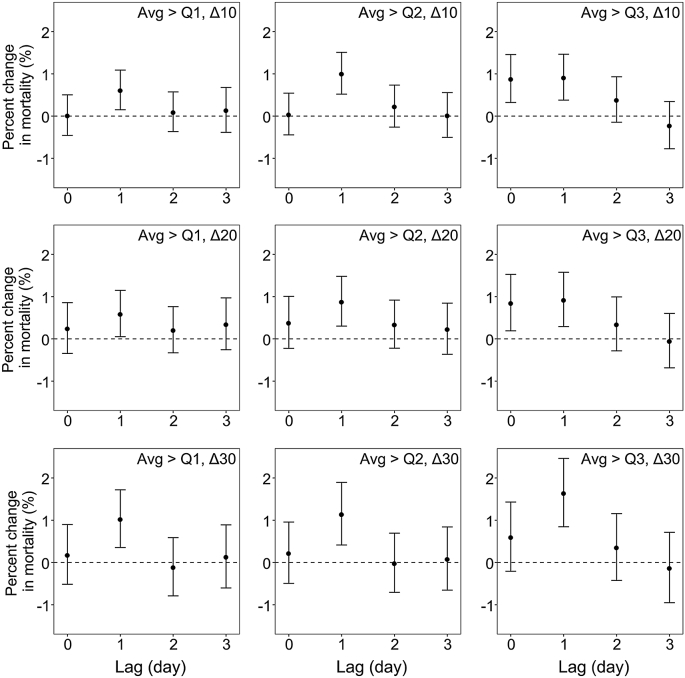
Percent changes in total cardiovascular disease mortality associated with different definitions of nocturnal ozone enhancement at multiple lags. Models were adjusted for time trend, temperature, relative humidity, day of the week, and maximum daily 8-h average ozone.

The associations of NOE with total CVD peaked at lag 1 d and then declined considerably or became insignificant at lag 2 or 3 d (Fig. 1). The magnitude of the associations varied by NOE definition, even at the same lag. On the day after NOE (lag 1 d), risks of total CVD mortality increased by 0.6%–1.7% (Table S4). More specifically, risk estimates appeared to be greater for a more strictly defined NOE. At lag 1 d, the risk increased by 1.7% (95% CI: 0.8%, 2.5%) for total CVD mortality under the strictest definition, versus 0.6% (95% CI: 0.2%, 1.1%) under the most lenient definition. Moreover, risk estimates depended on both nighttime O_3_ averages and peaks. For equivalent averages (“Avg > Q3”), total CVD mortality increased by 0.9 % (95% CI: 0.4%, 1.5%) and 1.7 % (95% CI: 0.8%, 2.5%), respectively, on the day after the NOE defined by “Δ10” and “Δ30”. For equivalent peak increment (“Δ30”), total CVD mortality increased by 1.0 % (95% CI: 0.4%, 1.7%) and 1.7% (95% CI: 0.8%, 2.5%), respectively, on the day after the NOE defined by “Avg > Q1” and “Avg > Q3”. Between-city heterogeneity was low, with *I*^2^ statistics ranging from 0.27% to 19.29% for total CVD mortality.

Then, we replaced the binary NOE variable with a continuous variable to assess the effect of nighttime O_3_ on total CVD mortality. The lag patterns for nighttime O_3_ were similar to those for NOE, with the highest risk estimates occurring at lag 1 d (Fig. S2). Specifically, a 10 μg/m^3^ increase in nighttime O_3_ concentrations was associated with a 0.1% (95% CI: 0.0%, 0.1%) increase in daily total CVD mortality at lag 1 d, which was slightly smaller than that for daytime O_3_ exposure.

### 3.3 Associations between NOE and cause-specific mortality

**Fig. 2:**
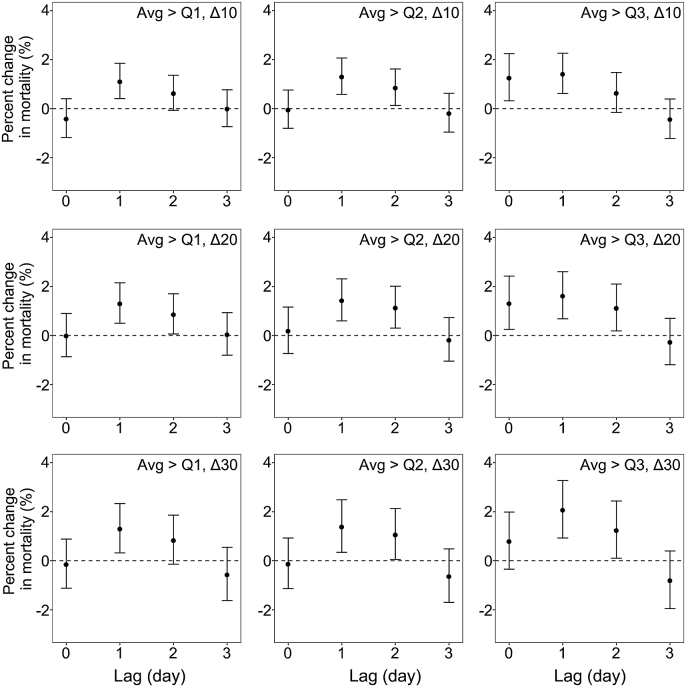
Percent changes in coronary heart disease mortality associated with different definitions of nocturnal ozone enhancement at multiple lags. Models were adjusted for time trend, temperature, relative humidity, day of the week, and maximum daily 8-h average ozone.

The lag patterns for the associations of NOE with CHD mortality were similar to those of total CVD (Fig. 2). On the day after NOE (lag 1 d), risks of CHD mortality increased by 1.1%–2.1% (Table S5). At lag 1 d, the risk increased by 2.1% (95% CI: 0.9%, 3.3%) for CHD mortality under the strictest definition, while the corresponding increase was 1.1% (95% CI: 0.4%, 1.9%) under the most lenient definition. The effects of NOE on CHD mortality were also influenced by both the averages and peaks of NOE. Between-city heterogeneity was low, with *I*^2^ statistics ranging from 1.21% to 19.58% for CHD mortality.

**Fig. 3:**
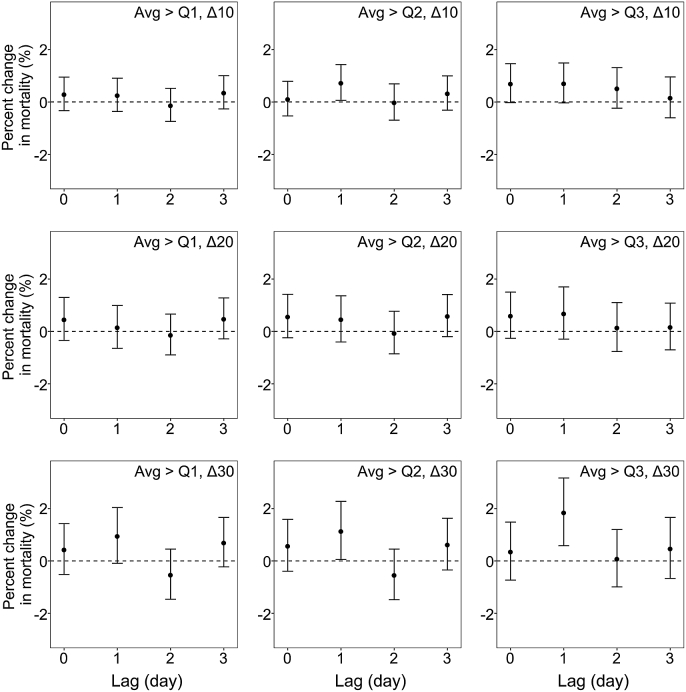
Percent changes in stroke mortality associated with different definitions of nocturnal ozone enhancement at multiple lags. Models were adjusted for time trend, temperature, relative humidity, day of the week, and maximum daily 8-h average ozone.

Although the lag patterns of the associations between NOE and stroke mortality varied by different NOE definitions, we also found larger effect estimates at lag 1 d (Fig. 3). Under the strictest definition, a 1.9% increase (95% CI: 0.6%, 3.2%) in stroke mortality was observed on the day after NOE (lag 1 d). Meanwhile, at lag 1 d, NOE was associated with increases of 0.7% (95% CI: 0.1%, 1.4%) and 1.2% (95% CI: 0.1%, 2.3%) in stroke mortality under the definitions of “Avg > Q2, Δ10” and “Avg > Q2, Δ30”, respectively. Associations between other defined NOE and stroke mortality were statistically insignificant (Table S6).

### 3.4 Sensitivity analyses

In sensitivity analyses (Table 2), the percent changes in mortality associated with the most strictly defined NOE at lag 1 d remained almost unchanged when single-day MDA8 O_3_ concentrations were replaced with either four-day MDA8 O_3_ concentrations or single-day 24-h average O_3_ concentrations, except for a slight decrease in stroke mortality when using 24-h average O_3_. These associations did not change appreciably when using three alternative approaches to adjust for the impacts of ambient temperature and further adjusting for PM_2.5_. We observed slight increases in effect estimates when changing the *df* for the spline of time trend from 6 to 5 or 7 per year. Finally, restricting the analysis to 69 cities with 3 years of data led to slight increases in the association between NOE and CHD mortality. The associations between other defined NOE and mortality were also robust to such adjustments (data not shown).

**Table 2:** Percent changes in mortality and 95% confidence intervals associated with the most strictly defined nocturnal ozone enhancement (lag 1 d).

	CVD	CHD	Stroke
Main model	1.6 (0.8, 2.5)	2.1 (0.9, 3.3)	1.9 (0.6, 3.2)
Alternative O_3_ adjustments			
LAG03_O_3_	1.7 (0.8, 2.5)	2.2 (1.0, 3.4)	2.0 (0.7, 3.3)
24h_O_3_	1.5 (0.8, 2.3)	2.2 (1.0, 3.3)	1.5 (0.2, 2.8)
Alternative temperature adjustments			
ns (LAG0_temp, 6)	1.6 (0.8, 2.4)	2.0 (0.8, 3.2)	1.8 (0.5, 3.1)
DLNM_temp	1.5 (0.7, 2.3)	1.9 (0.7, 3.1)	1.8 (0.5, 3.1)
DLNM_temp + extreme heat	1.5 (0.7, 2.3)	1.9 (0.7, 3.1)	1.8 (0.5, 3.1)
Additional PM_2.5_ adjustments			
Main model + LAG03_PM_2.5_	1.6 (0.7, 2.5)	2.1 (0.9, 3.3)	1.8 (0.5, 3.2)
Model with DLNM_temp + LAG03_PM_2.5_	1.5 (0.6, 2.3)	1.8 (0.6, 3.1)	1.8 (0.5, 3.1)
Alternative *df* for time trend			
5 per year	1.8 (0.9, 2.6)	2.3 (1.1, 3.5)	2.0 (0.7, 3.4)
7 per year	1.7 (0.8, 2.5)	2.3 (1.1, 3.4)	1.8 (0.5, 3.2)
Restricting to cities with 3 years of data			
N = 69 cities	1.6 (0.7, 2.6)	2.3 (1.0, 3.7)	1.8 (0.3, 3.4)

Notes: Main model was adjusted for time trend, temperature, relative humidity, day of the week, and maximum daily 8-h average O_3_. O_3_, ozone; PM_2.5_, particulate matter with an aerodynamic diameter less than or equal to 2.5 μm; CVD, cardiovascular disease; CHD, coronary heart disease; *df*, degrees of freedom.

### 3.5 Stratified analyses

**Fig. 4:**
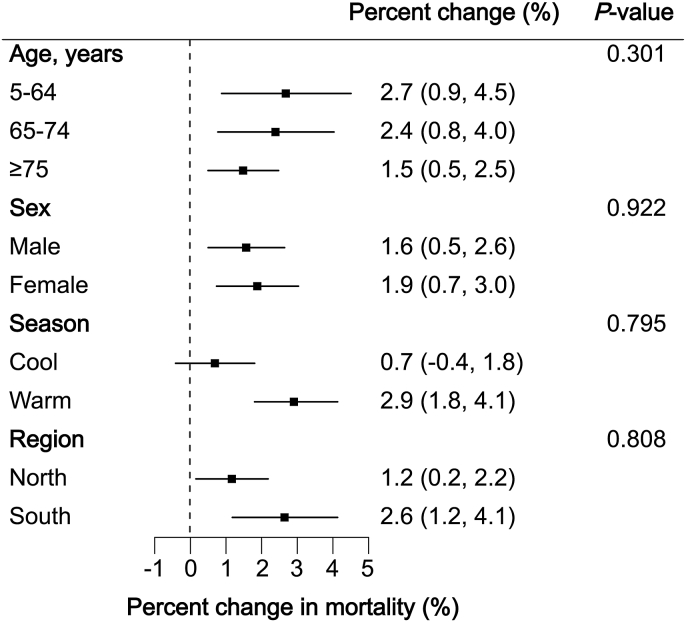
Percent changes in total cardiovascular disease mortality associated with the most strictly defined nocturnal ozone enhancement (lag 1 d), stratified by age, sex, season, and region. The warm season was defined as May through October and the cool season as November through April. The southern and northern regions were divided based on the Qinling-Huaihe Line. *P*-values indicate the statistical significance of between-group differences. Models were adjusted for time trend, temperature and relative humidity, day of the week, and maximum daily 8-h average ozone.

Fig. 4 presents the impacts of NOE (using the strictest definition) on total CVD mortality, stratified by individual characteristics, season, and region. The associations were relatively stronger for people aged 5–64 years, females, during the warm season, and the southern region, as manifested by larger risk estimates and statistical significance. In contrast, smaller effect estimates were observed for those aged 65 years and older, males, the cool season, and the northern region. However, none of these between-group differences reached statistical significance.

## 4 Discussion

We observed significant positive associations between multiple definitions of NOE and mortality from total CVD and CHD, with the strongest association on the day after NOE, depending on both O_3_ averages and peaks on NOE days. These associations remained robust after adjustment for PM_2.5_, daytime O_3_ exposure, temperature, and long-term trends, and restricting the analyses to cities with 3 years of data. Although stratified analyses did not reveal statistically significant differences between subgroups, relatively stronger associations were observed for individuals aged 5–64 years, females, during the warm season, and in the southern region. In this study, we did not find any significant associations between NOE and stroke mortality.

Our study demonstrated the adverse effects of NOE on total CVD mortality. These effects remained stable after adjusting for MDA8 and 24-h average O_3_ concentration, suggesting that the health risks posed by rapid nighttime O_3_ increases may be independent of daytime or average O_3_ exposure. With accelerating urbanization and climate change, the frequency of NOE events is projected to increase [26]. In this context, public health policies should focus on addressing nighttime O_3_ pollution, particularly in NOE-prone areas. In contrast to the extensive research on daytime O_3_ (e.g., MDA8) [27,28], no prior studies have specifically investigated the health effects of NOE, leaving our findings without direct precedent. However, our additional analysis revealed a significant association between nighttime average O_3_ concentrations and total CVD mortality. This further demonstrates that despite lower nighttime O_3_ concentrations, exposure during this period remains a significant public health concern. It should be noted that using nighttime average O_3_ concentrations yields weaker adverse effects than NOE. A time-series study conducted in a Chinese city even failed to find a significant association between nighttime average O_3_ concentrations and daily mortality [29]. This suggests that NOE events might be a more appropriate indicator to capture the health hazards of nighttime O_3_ air pollution compared to average concentrations, as NOE events were defined based on both averages and rapid increases in O_3_ concentrations. Controlled human exposure studies also found that peak exposures (triangular profile) posed a greater health hazard than steady exposures (square-wave profile) at equivalent average concentrations [11,12], which partially supports our finding. Meanwhile, we noted that the effects of nighttime O_3_ average exposure were slightly smaller than daytime O_3_ exposure, which may be due to lower nighttime concentrations and the predominance of parasympathetic activity [16,17,30], facilitating cardiac recovery during the night.

Our findings revealed a short delay between NOE days and cardiovascular deaths, suggesting lagged effects of NOE. This observation was consistent with most previous studies that documented lagged effects of air pollutants on health [[31], [32], [33], [34]]. Moreover, this finding is biologically plausible. First, the generation of reactive oxygen species induced by low-level O_3_ exposure at night may require time to accumulate before initiating downstream biological processes [35,36]. Second, the cardiovascular effects of O_3_ may be mediated by a systemic inflammatory cascade triggered by pulmonary oxidative stress, a process that also takes time to develop [36,37]. Third, research has found that O_3_ can induce hypoxia and compensatory hematological responses one day post-exposure, potentially exacerbating cardiovascular damage [34]. Our analysis also demonstrated that the health impact of NOE was influenced by both nighttime average O_3_ concentrations on NOE days and peak increments during NOE events. This dual influence suggests that comprehensive NOE definitions should take these two dimensions into account to better assess their health risks. From a policy standpoint, these findings imply that effective mitigation of nocturnal O_3_ pollution may require regulatory standards that consider not only average concentrations but also short-term peak concentrations.

Further analysis showed a stronger association of NOE with CHD mortality and a weaker association with stroke mortality, suggesting that CHD may be the primary driver of NOE-induced overall cardiovascular mortality. This finding aligns with previous research that has consistently demonstrated stronger associations between O_3_ exposure and CHD mortality compared to stroke mortality [38]. The distinct pathophysiological mechanisms of CHD and stroke may account for these differences. O_3_ has been demonstrated to induce systemic inflammatory responses and oxidative stress, impair vascular function, and cause endothelial damage, thereby promoting the progression of atherosclerosis, particularly in the coronary arteries [8,39,40], which are closely associated with the development of CHD. In contrast, stroke encompasses multiple subtypes (e.g., ischemic and hemorrhagic) with heterogeneous etiologies and pathophysiological pathways [41]. The diversity of stroke subtypes and their multifactorial etiologies may dilute the overall association between NOE and stroke mortality.

Although the effect modification by age and sex did not reach statistical significance, our results suggest that relatively younger people and females may be more susceptible to NOE-associated total CVD mortality. Younger individuals may experience increased NOE exposure due to greater engagement in nighttime activities, such as working and socializing [42]. The slightly stronger association among females is consistent with previous studies, which may be related to anatomical differences and more potent inflammatory and immune reactions in females compared to males [43]. In addition, we found that the association between NOE days and total CVD mortality appeared to be stronger in the southern region and during the warm season. This geographical and seasonal pattern may be explained by enhanced ventilation during warmer conditions that may enhance NOE exposure [8,26], and synergistic effects between O_3_ and heat stress that may amplify NOE’s health impacts [8].

This study has several notable strengths, including a large population sample, extensive geographic coverage, and the application of multiple NOE definitions. To the best of our knowledge, this is the first epidemiological investigation to estimate health effects associated with NOE events. However, certain limitations should be acknowledged. First, since our analysis was limited to 2013–2015, these findings may not be directly extrapolated to the current situation, in which nocturnal O_3_ concentrations are rising. Second, similar to previous time-series studies, we relied on outdoor concentrations as exposure proxies, which may introduce exposure misclassification since people generally stay indoors at night [[44], [45], [46]]. However, previous studies have shown that although indoor O_3_ levels are lower, they still vary with outdoor concentrations, suggesting that people can still be influenced by fluctuations in outdoor O_3_ concentrations when staying indoors [47,48]. Moreover, when people are indoors, they may be directly exposed to O_3_ oxidation products from indoor surfaces and human skin [47,49]. Importantly, such exposure misclassification is likely non-differential and may bias the effect estimates toward the null [50,51]. Third, by focusing on city-level NOE days, we may have overlooked localized or single-site NOE events, thereby failing to capture their health impacts on finer spatial scales. Fourth, although we specified the exposure window on the day of death to maximize the likelihood that deaths occurred after NOE exposure, we cannot completely rule out reverse temporality due to the lack of the exact time of death. However, previous studies have shown that sudden cardiac deaths most commonly occur after 06:00 a.m. [13,52,53], suggesting that the possibility of death occurring prior to exposure is low. Moreover, our findings indicated that the NOE event occurring the day before death (lag 1 day) had a more significant impact on mortality. Despite these limitations, the large population sample helps mitigate some of these concerns. Future research should address these limitations by expanding the time coverage, improving exposure assessments, and incorporating individual-level data to better assess the health effects of NOE events.

In conclusion, our study demonstrated that NOE events were significantly associated with increased risks of total CVD and CHD mortality, providing new insights into the health impacts of O_3_ exposure. These results underscore the necessity of addressing nocturnal O_3_ pollution as an emerging public health concern, and policymakers should implement time-phased control measures to mitigate the health risks posed by both daytime and nighttime O_3_ exposure.

## CRediT authorship contribution statement

**Zhihan Jian:** Writing – review & editing, Writing – original draft, Visualization, Formal analysis. **Peng Yin:** Visualization, Formal analysis, Data curation. **Renjie Chen:** Writing – review & editing, Supervision. **Lijun Wang:** Writing – original draft, Data curation. **Yixiang Zhu:** Formal analysis, Data curation. **Xia Meng:** Writing – review & editing, Data curation. **Haidong Kan:** Writing – review & editing, Supervision, Conceptualization. **Yue Niu:** Writing – review & editing, Supervision, Conceptualization, Funding acquisition. **Maigeng Zhou:** Writing – review & editing, Supervision, Conceptualization.

## Declaration of competing interests

The authors declare no competing interests.
