# Supplementary material for: Association between nocturnal ozone enhancement and daily cardiovascular mortality: A multi-city study in China
Source: Eco Environ Health. 2025 Dec 24;5(1):100211. doi: 10.1016/j.eehl.2025.100211 (PMC12816898; doi:10.1016/j.eehl.2025.100211)
Supplement: Multimedia component 1 [file mmc1.docx]

**Supplementary Information**

**Association between nocturnal ozone enhancement and daily cardiovascular mortality: A multi-city study in China**

Zhihan Jian^a,1^, Peng Yin^b,1^, Renjie Chen^a^, Lijun Wang^b^, Yixiang Zhu^a^, Xia Meng^a^, Haidong Kan^a^, Yue Niu^a,*^, Maigeng Zhou^b,*^

***** **Corresponding authors.**

^1^ Co-first authors.

**Table S1.** Descriptive statistics of daily deaths and air pollutant levels in 272 Chinese cities from 2013 to 2015

| Variables | Mean | SD | Min | P_25_ | Median | P_75_ | Max |
| --- | --- | --- | --- | --- | --- | --- | --- |
| Deaths |  |  |  |  |  |  |  |
| CVD | 8 | 7 | 1 | 3 | 6 | 10 | 65 |
| CHD | 3 | 3 | 0 | 1 | 2 | 3 | 28 |
| Stroke | 4 | 4 | 0 | 2 | 3 | 5 | 33 |
| Air pollutants (μg/m³) |  |  |  |  |  |  |  |
| PM_2.5_ (24-h) | 56 | 20 | 18 | 41 | 54 | 67 | 127 |
| Ozone (8-h) | 77 | 14 | 36 | 68 | 77 | 87 | 113 |

SD, standard deviation; Min, minimum; P_25_, 25th percentile value; P_75_, 75th percentile value; Max, maximum; CVD, cardiovascular disease; CHD, coronary heart disease; PM_2.5_, particulate matter with an aerodynamic diameter less than or equal to 2.5 μm.

**Table S2.** Definitions and numbers of days with nocturnal ozone enhancement at the city level in 272 Chinese cities in different seasons from 2013 to 2015

| Definition | Description | | Number of days with NOE per year | | | | | | | Total number of days with NOE |
| --- | --- | --- | --- | --- | --- | --- | --- | --- | --- | --- |
|  | Average (Avg) | Difference (Δ) | Mean | SD | Min | P_25_ | Median | P_75_ | Max |  |
| Cool |  |  |  |  |  |  |  |  |  |  |
| Avg > Q1, Δ10 | > quartile 1 | > 10 μg/m³ | 72 | 25 | 7 | 56 | 67 | 90 | 129 | 35,011 |
| Avg > Q1, Δ20 | > quartile 1 | > 20 μg/m³ | 39 | 24 | 0 | 20 | 36 | 51 | 126 | 18,613 |
| Avg > Q1, Δ30 | > quartile 1 | > 30 μg/m³ | 22 | 20 | 0 | 7 | 16 | 30 | 123 | 10,437 |
| Avg > Q2, Δ10 | > quartile 2 | > 10 μg/m³ | 52 | 16 | 3 | 41 | 49 | 64 | 87 | 25,217 |
| Avg > Q2, Δ20 | > quartile 2 | > 20 μg/m³ | 30 | 17 | 0 | 18 | 29 | 40 | 84 | 14,405 |
| Avg > Q2, Δ30 | > quartile 2 | > 30 μg/m³ | 18 | 15 | 0 | 6 | 14 | 25 | 84 | 8,444 |
| Avg > Q3, Δ10 | > quartile 3 | > 10 μg/m³ | 29 | 8 | 2 | 23 | 27 | 35 | 46 | 13,953 |
| Avg > Q3, Δ20 | > quartile 3 | > 20 μg/m³ | 18 | 9 | 0 | 11 | 17 | 23 | 44 | 8,567 |
| Avg > Q3, Δ30 | > quartile 3 | > 30 μg/m³ | 11 | 8 | 0 | 5 | 9 | 16 | 42 | 5,321 |
| Warm |  |  |  |  |  |  |  |  |  |  |
| Avg > Q1, Δ10 | > quartile 1 | > 10 μg/m³ | 79 | 29 | 1 | 61 | 80 | 100 | 134 | 40,764 |
| Avg > Q1, Δ20 | > quartile 1 | > 20 μg/m³ | 41 | 30 | 0 | 18 | 33 | 64 | 132 | 21,205 |
| Avg > Q1, Δ30 | > quartile 1 | > 30 μg/m³ | 22 | 24 | 0 | 5 | 12 | 32 | 128 | 11,189 |
| Avg > Q2, Δ10 | > quartile 2 | > 10 μg/m³ | 57 | 18 | 1 | 45 | 58 | 71 | 89 | 29,414 |
| Avg > Q2, Δ20 | > quartile 2 | > 20 μg/m³ | 32 | 21 | 0 | 16 | 27 | 48 | 89 | 16,496 |
| Avg > Q2, Δ30 | > quartile 2 | > 30 μg/m³ | 18 | 17 | 0 | 4 | 11 | 27 | 85 | 9,133 |
| Avg > Q3, Δ10 | > quartile 3 | > 10 μg/m³ | 32 | 9 | 1 | 27 | 32 | 39 | 47 | 16,458 |
| Avg > Q3, Δ20 | > quartile 3 | > 20 μg/m³ | 19 | 12 | 0 | 11 | 18 | 28 | 47 | 10,004 |
| Avg > Q3, Δ30 | > quartile 3 | > 30 μg/m³ | 11 | 10 | 0 | 3 | 8 | 18 | 46 | 5,888 |

The warm season is defined as May through October and the cool season is defined as November through April.

NOE, nocturnal ozone enhancement; Max, maximum; Min, minimum; SD, standard deviation; P_25_, 25th percentile value; P_75_, 75th percentile value.

**Table S3.** Definitions and numbers of days with nocturnal ozone enhancement at the city level in 272 Chinese cities in different regions from 2013 to 2015

| Definition | Description | | Number of days with NOE per year | | | | | | | Total number of days with NOE |
| --- | --- | --- | --- | --- | --- | --- | --- | --- | --- | --- |
|  | Average (Avg) | Difference (Δ) | Mean | SD | Min | P_25_ | Median | P_75_ | Max |  |
| North |  |  |  |  |  |  |  |  |  |  |
| Avg > Q1, Δ10 | > quartile 1 | > 10 μg/m³ | 171 | 45 | 62 | 141 | 164 | 205 | 261 | 41,139 |
| Avg > Q1, Δ20 | > quartile 1 | > 20 μg/m³ | 110 | 50 | 20 | 78 | 106 | 134 | 258 | 26,486 |
| Avg > Q1, Δ30 | > quartile 1 | > 30 μg/m³ | 68 | 46 | 7 | 38 | 63 | 86 | 251 | 16,212 |
| Avg > Q2, Δ10 | > quartile 2 | > 10 μg/m³ | 120 | 29 | 46 | 99 | 119 | 142 | 176 | 28,865 |
| Avg > Q2, Δ20 | > quartile 2 | > 20 μg/m³ | 83 | 34 | 18 | 62 | 80 | 102 | 173 | 19,830 |
| Avg > Q2, Δ30 | > quartile 2 | > 30 μg/m³ | 54 | 33 | 7 | 34 | 51 | 66 | 169 | 12,807 |
| Avg > Q3, Δ10 | > quartile 3 | > 10 μg/m³ | 65 | 15 | 23 | 53 | 64 | 77 | 93 | 15,674 |
| Avg > Q3, Δ20 | > quartile 3 | > 20 μg/m³ | 48 | 18 | 10 | 37 | 47 | 58 | 89 | 11,420 |
| Avg > Q3, Δ30 | > quartile 3 | > 30 μg/m³ | 34 | 18 | 3 | 22 | 33 | 42 | 88 | 7,876 |
| South |  |  |  |  |  |  |  |  |  |  |
| Avg > Q1, Δ10 | > quartile 1 | > 10 μg/m³ | 132 | 48 | 8 | 101 | 131 | 162 | 239 | 34,636 |
| Avg > Q1, Δ20 | > quartile 1 | > 20 μg/m³ | 52 | 34 | 1 | 25 | 45 | 68 | 170 | 13,332 |
| Avg > Q1, Δ30 | > quartile 1 | > 30 μg/m³ | 21 | 18 | 0 | 7 | 15 | 29 | 97 | 5,414 |
| Avg > Q2, Δ10 | > quartile 2 | > 10 μg/m³ | 99 | 32 | 4 | 77 | 98 | 121 | 166 | 25,766 |
| Avg > Q2, Δ20 | > quartile 2 | > 20 μg/m³ | 43 | 26 | 1 | 24 | 39 | 57 | 123 | 11,071 |
| Avg > Q2, Δ30 | > quartile 2 | > 30 μg/m³ | 18 | 15 | 0 | 7 | 14 | 26 | 72 | 4,770 |
| Avg > Q3, Δ10 | > quartile 3 | > 10 μg/m³ | 56 | 17 | 3 | 45 | 55 | 68 | 89 | 14,737 |
| Avg > Q3, Δ20 | > quartile 3 | > 20 μg/m³ | 28 | 16 | 1 | 16 | 26 | 38 | 69 | 7,151 |
| Avg > Q3, Δ30 | > quartile 3 | > 30 μg/m³ | 13 | 10 | 0 | 5 | 10 | 18 | 46 | 3,333 |

The southern and northern regions were divided based on the Qinling–Huaihe Line.

NOE, nocturnal ozone enhancement; Max, maximum; Min, minimum; SD, standard deviation; P_25_, 25th percentile value; P_75_, 75th percentile value.

**Table S4.** Percent changes in total cardiovascular disease mortality and 95% confidence intervals associated with different definitions of nocturnal ozone enhancement at multiple lags

| Definition | Lag 0 d | Lag 1 d | Lag 2 d | Lag 3 d |
| --- | --- | --- | --- | --- |
| Avg > Q1, Δ10 | 0.0 (-0.5, 0.5) | 0.6 (0.2, 1.1) | 0.1 (-0.4, 0.6) | 0.1 (-0.4, 0.7) |
| Avg > Q1, Δ20 | 0.3 (-0.3, 0.9) | 0.6 (0.1, 1.1) | 0.2 (-0.3, 0.8) | 0.4 (-0.3, 1.0) |
| Avg > Q1, Δ30 | 0.2 (-0.5, 0.9) | 1.0 (0.4, 1.7) | -0.1 (-0.8, 0.6) | 0.1 (-0.6, 0.9) |
| Avg > Q2, Δ10 | 0.0 (-0.4, 0.5) | 1.0 (0.5, 1.5) | 0.2 (-0.3, 0.7) | 0.0 (-0.5, 0.6) |
| Avg > Q2, Δ20 | 0.4 (-0.2, 1.0) | 0.9 (0.3, 1.5) | 0.3 (-0.2, 0.9) | 0.2 (-0.4, 0.8) |
| Avg > Q2, Δ30 | 0.2 (-0.5, 1.0) | 1.2 (0.4, 1.9) | 0.0 (-0.7, 0.7) | 0.1 (-0.7, 0.8) |
| Avg > Q3, Δ10 | 0.9 (0.3, 1.5) | 0.9 (0.4, 1.5) | 0.4 (-0.1, 0.9) | -0.2 (-0.8, 0.3) |
| Avg > Q3, Δ20 | 0.9 (0.2, 1.5) | 0.9 (0.3, 1.6) | 0.4 (-0.3, 1.0) | 0.0 (-0.7, 0.6) |
| Avg > Q3, Δ30 | 0.6 (-0.2, 1.4) | 1.7 (0.8, 2.5) | 0.4 (-0.4, 1.2) | -0.1 (-0.9, 0.7) |

Models were adjusted for time trend, temperature and relative humidity, day of the week, and maximum daily 8-hour average ozone.

**Table S5.** Percent changes in coronary heart disease mortality and 95% confidence intervals associated with different definitions of nocturnal ozone enhancement events at multiple lags

| Definition | Lag 0 d | Lag 1 d | Lag 2 d | Lag 3 d |
| --- | --- | --- | --- | --- |
| Avg > Q1, Δ10 | -0.4 (-1.2, 0.4) | 1.1 (0.4, 1.9) | 0.6 (-0.1, 1.4) | 0.0 (-0.7, 0.8) |
| Avg > Q1, Δ20 | 0.0 (-0.9, 0.9) | 1.3 (0.5, 2.2) | 0.9 (0.1, 1.7) | 0.1 (-0.8, 0.9) |
| Avg > Q1, Δ30 | -0.1 (-1.1, 0.9) | 1.3 (0.3, 2.3) | 0.9 (-0.1, 1.9) | -0.5 (-1.6, 0.5) |
| Avg > Q2, Δ10 | 0.0 (-0.8, 0.8) | 1.3 (0.6, 2.1) | 0.9 (0.1, 1.6) | -0.2 (-1.0, 0.6) |
| Avg > Q2, Δ20 | 0.2 (-0.7, 1.2) | 1.5 (0.6, 2.3) | 1.2 (0.3, 2.0) | -0.2 (-1.0, 0.7) |
| Avg > Q2, Δ30 | -0.1 (-1.1, 0.9) | 1.4 (0.3, 2.5) | 1.1 (0.1, 2.1) | -0.6 (-1.7, 0.5) |
| Avg > Q3, Δ10 | 1.3 (0.3, 2.2) | 1.4 (0.6, 2.3) | 0.7 (-0.2, 1.5) | -0.4 (-1.2, 0.4) |
| Avg > Q3, Δ20 | 1.3 (0.2, 2.4) | 1.6 (0.7, 2.6) | 1.1 (0.2, 2.1) | -0.2 (-1.2, 0.7) |
| Avg > Q3, Δ30 | 0.8 (-0.3, 2.0) | 2.1 (0.9, 3.3) | 1.3 (0.1, 2.4) | -0.8 (-2.0, 0.4) |

Models were adjusted for time trend, temperature and relative humidity, day of the week, and maximum daily 8-hour average ozone.

**Table S6.** Percent changes in stroke mortality and 95% confidence intervals associated with different definitions of nocturnal ozone enhancement events at multiple lags

| Definition | Lag 0 d | Lag 1 d | Lag 2 d | Lag 3 d |
| --- | --- | --- | --- | --- |
| Avg > Q1, Δ10 | 0.3 (-0.3, 1.0) | 0.3 (-0.4, 0.9) | -0.1 (-0.7, 0.5) | 0.4 (-0.3, 1.0) |
| Avg > Q1, Δ20 | 0.5 (-0.3, 1.3) | 0.2 (-0.6, 1.0) | -0.1 (-0.9, 0.7) | 0.5 (-0.3, 1.3) |
| Avg > Q1, Δ30 | 0.5 (-0.5, 1.4) | 1.0 (-0.1, 2.0) | -0.5 (-1.5, 0.4) | 0.7 (-0.2, 1.7) |
| Avg > Q2, Δ10 | 0.1 (-0.5, 0.8) | 0.7 (0.1, 1.4) | 0.0 (-0.7, 0.7) | 0.3 (-0.3, 1.0) |
| Avg > Q2, Δ20 | 0.6 (-0.2, 1.4) | 0.5 (-0.4, 1.4) | 0.0 (-0.9, 0.8) | 0.6 (-0.2, 1.4) |
| Avg > Q2, Δ30 | 0.6 (-0.4, 1.6) | 1.2 (0.1, 2.3) | -0.5 (-1.5, 0.4) | 0.6 (-0.3, 1.6) |
| Avg > Q3, Δ10 | 0.7 (0.0, 1.5) | 0.7 (0.0, 1.5) | 0.5 (-0.2, 1.3) | 0.2 (-0.6, 1.0) |
| Avg > Q3, Δ20 | 0.6 (-0.3, 1.5) | 0.7 (-0.3, 1.7) | 0.2 (-0.8, 1.1) | 0.2 (-0.7, 1.1) |
| Avg > Q3, Δ30 | 0.4 (-0.7, 1.5) | 1.9 (0.6, 3.2) | 0.1 (-1.0, 1.2) | 0.5 (-0.7, 1.7) |

Models were adjusted for time trend, temperature and relative humidity, day of the week, and maximum daily 8-hour average ozone.


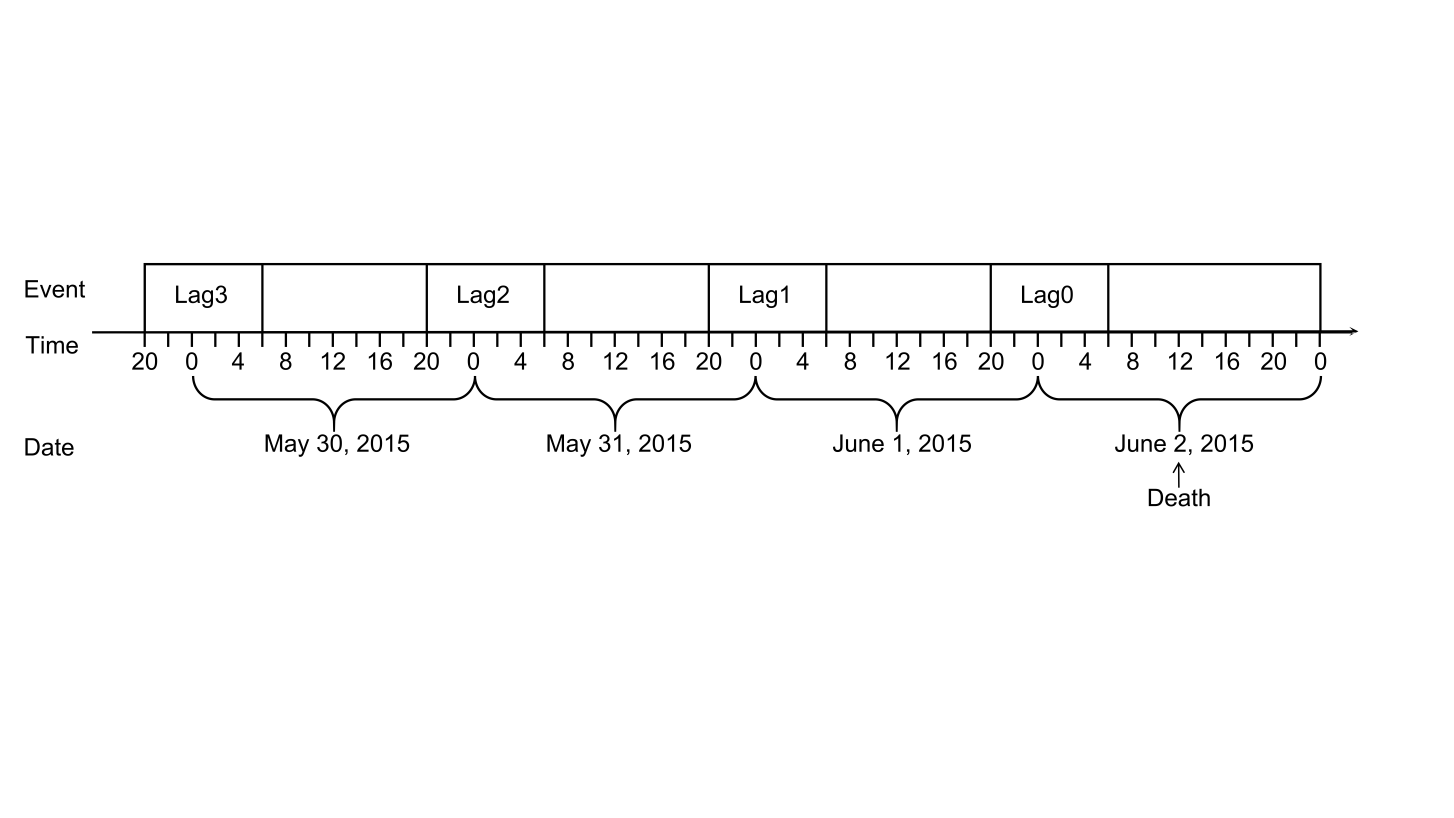


**Figure S1.** Definition for nocturnal ozone enhancement day at different single-day lags


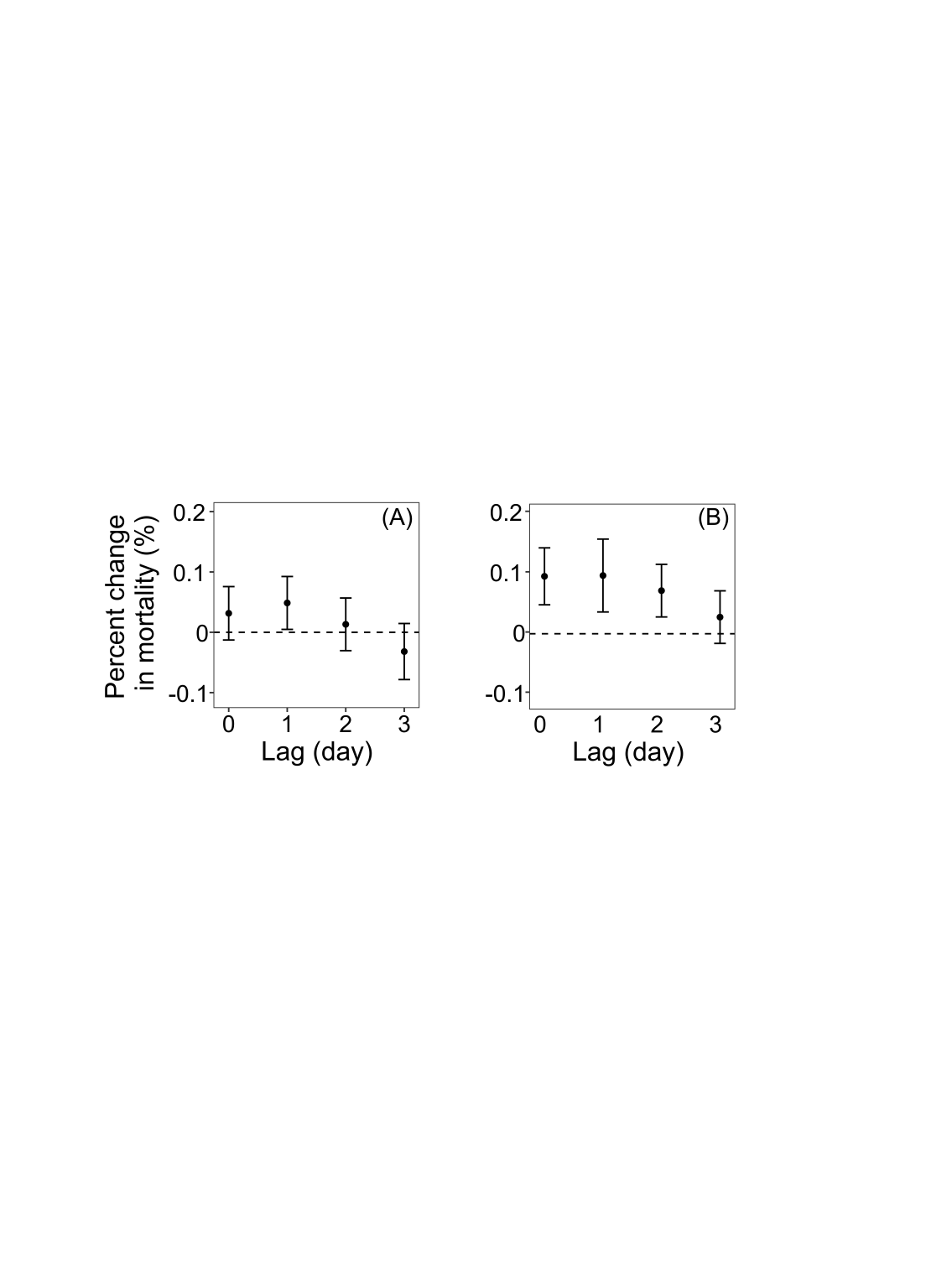


**Figure S2.** Percent changes in total cardiovascular disease mortality per 10 μg/m^3^ increase in nighttime (A) and daytime (B) average ozone concentrations at multiple lags

Nighttime average ozone concentration was defined as the average ozone concentration between 8:00 p.m. to 6:00 a.m. Daytime average ozone concentration was defined as the average ozone concentration between 10:00 a.m. and 6:00 p.m. (i.e., maximum daily 8-hour average concentrations). Nighttime and daytime ozone concentrations were incorporated into models simultaneously, with adjustments for time trend, temperature and relative humidity, and day of the week.
